# Defining NASH from a Multi-Omics Systems Biology Perspective

**DOI:** 10.3390/jcm10204673

**Published:** 2021-10-12

**Authors:** Lili Niu, Karolina Sulek, Catherine G. Vasilopoulou, Alberto Santos, Nicolai J. Wewer Albrechtsen, Simon Rasmussen, Florian Meier, Matthias Mann

**Affiliations:** 1 Novo Nordisk Foundation Center for Protein Research, Faculty of Health and Medical Sciences, University of Copenhagen, 2200 Copenhagen, Denmark; karolina.sulek@regionh.dk (K.S.); alberto.santos@sund.ku.dk (A.S.); nicolai.albrechtsen@sund.ku.dk (N.J.W.A.); simon.rasmussen@cpr.ku.dk (S.R.); mmann@biochem.mpg.de (M.M.); 2 Department of Proteomics and Signal Transduction, Max Planck Institute of Biochemistry, 82152 Martinsried, Germany; vasilopoulou@biochem.mpg.de (C.G.V.); Florian.Meier@med.uni-jena.de (F.M.); 3 Systems Medicine, Steno Diabetes Center Copenhagen, 2820 Gentofte, Denmark; 4 Center for Health Data Science, University of Copenhagen, 2200 Copenhagen, Denmark; 5 Big Data Institute, Nuffield Department of Medicine, University of Oxford, Oxford OX3 7LF, UK; 6 Department of Clinical Biochemistry, Rigshospitalet, 2100 Copenhagen, Denmark; 7 Department of Biomedical Sciences, Faculty of Health and Medical Sciences, University of Copenhagen, 2200 Copenhagen, Denmark; 8 Functional Proteomics, Jena University Hospital, 07747 Jena, Germany

**Keywords:** biomarker discovery, liver disease, machine learning, multi-omics, NAFLD, systems biology

## Abstract

Non-alcoholic steatohepatitis (NASH) is a chronic liver disease affecting up to 6.5% of the general population. There is no simple definition of NASH, and the molecular mechanism underlying disease pathogenesis remains elusive. Studies applying single omics technologies have enabled a better understanding of the molecular profiles associated with steatosis and hepatic inflammation—the commonly accepted histologic features for diagnosing NASH, as well as the discovery of novel candidate biomarkers. Multi-omics analysis holds great potential to uncover new insights into disease mechanism through integrating multiple layers of molecular information. Despite the technical and computational challenges associated with such efforts, a few pioneering studies have successfully applied multi-omics technologies to investigate NASH. Here, we review the most recent technological developments in mass spectrometry (MS)-based proteomics, metabolomics, and lipidomics. We summarize multi-omics studies and emerging omics biomarkers in NASH and highlight the biological insights gained through these integrated analyses.

## 1. Introduction

In the past decade, high-throughput omics technologies have revolutionized biomedical research [1]. Obtaining multiple layers of molecular measurements such as genomics, transcriptomics, proteomics, metabolomics, and lipidomics helps to systematically understand health and disease states, and may uncover new biological insights into disease mechanisms. NASH is a severe form of non-alcoholic fatty liver disease (NAFLD) which may progress to irreversible end-stage liver disease (cirrhosis). It is also associated with an increased risk of complications from cardiovascular disease and kidney disease [2]. However, diagnostics and therapeutics are limited. Currently, NASH can only be diagnosed by pathological evaluation of liver biopsy, and is defined by the presence of excessive fat deposition in the liver exceeding 5% of hepatocytes, hepatocyte ballooning, and lobular inflammation, with or without fibrosis [3]. The pathogenesis of NASH has not been fully elucidated [4]. A “two-hit” hypothesis has been proposed in which “liver steatosis”, the “first hit” increases the susceptibility to NASH through a “second hit” such as endoplasmic reticulum and oxidative stress [5,6]. There are no markers with sufficient sensitivity and accuracy for the clinical use of non-invasive diagnosis of NASH [7,8]. Genome-wide association studies (GWAS) have identified robust and reproducible *loci* that contribute to NAFLD pathogenesis and variability of prognosis, including the non-synonymous single nucleotide polymorphisms (SNPs) in *PNPLA3* (phospholipase domain-containing 3), *TM6SF2* (transmembrane 6 superfamily member 2), *MBOAT7* (membrane-bound O-acyltransferase domain-containing protein 7), GCKR (glucokinase regulator), and *HSD17B13* (17-beta hydroxysteroid dehydrogenase 13) [9]. While the heritability estimates of NAFLD range from 20–70% in population, family-based, or twin studies, the proportion of heritability explained by known risk variants is still a modest 10–20% [10,11,12]. In addition, GWAS alone does not suffice to elucidate the functional roles of the identified genetic variation in disease onset and progression [13].

Regulation of gene expression gives rise to different cell types as determined by their transcriptional states, and therefore represents a pivotal link between genetic structure and the molecular phenotype. Transcriptomics can quantify up to tens of thousands of transcripts in cells or tissues and has been included in many routine biological studies. Single-cell RNA sequencing has identified 20 discrete resident cell populations in human liver providing an in-depth map of the human hepatic immune microenvironment [14]. A recent study applied bulk RNA sequencing to a group of 206 NAFLD patients, and identified gene expression signatures associated with early stages and stepwise progression of the disease [15]. Integration with publicly available single-cell RNAseq data allowed the authors to further dissect the likely relative contribution of specific intrahepatic cell populations to NAFLD pathogenesis and progression. These results showed that changes in the transcriptome represent potential clinically relevant markers of disease progression [15]. 

While transcriptomics gives a rough estimate of the expression level of transcripts into proteins, proteomics confirms the presence of proteins and provides direct measurements of their quantity and modification status, so it is closer to disease phenotype. Therefore, the study of protein profiles (proteomics) is integral to many research fields including biomarker discovery, drug development, and elucidation of disease mechanisms [16,17]. Metabolomics and its sub-field lipidomics are the most downstream members of the omics family. Despite the rapid progress in the field, the overwhelming chemical complexity and diversity of small biomolecules still pose great challenges to identification and quantification strategies and downstream bioinformatics analysis. Nevertheless, lipidomics is a very important technology in the study of NAFLD. Several lipid classes have been linked to lipotoxicity and progression of the disease [18,19]. Finally, an increasing number of studies applying multi-omics technologies to generate “big data” are being performed to address the pathophysiology and diagnostics of NASH. 

In this review, we focus on the technological aspects of mass spectrometry (MS)-based omics and the integrated application of omics in NASH research. In the first of three sections, we describe MS-based proteomics, metabolomics, and lipidomics technologies with a focus on state-of-the-art technical workflows. This is followed by highlights from recent proteomics studies and a systematic literature review describing metabolomics and lipidomics studies in NASH. Finally, we summarize the existing literature on emerging omics biomarkers and the application of multi-omics to NASH research. 

## 2. State-of-the-Art Proteomics, Metabolomics, and Lipidomics Technologies

### 2.1. MS-Based Proteomics

While genome sequencing deciphers the blueprint of human life, which is mostly static, the human proteome is a highly dynamic entity in terms of both number of proteoforms, their copy numbers, and their spatiotemporal expression. On top of the approximately 20,000 human protein-coding genes, a single protein-coding gene can easily produce as many as 100 proteoforms, including products of alternative splicing, those containing single amino acid polymorphisms arising from non-synonymous SNPs, and those carrying post-translational modifications (PTMs) [20,21].

In MS-based proteomics, “bottom-up” (or “shotgun”) proteomics is the most widely used workflow, in which proteins are subjected to proteolytic cleavage, and the resulting peptides are analyzed by liquid chromatography coupled online to tandem mass spectrometry (LC-MS/MS) [22,23,24]. Peptide identification relies on tandem MS/MS spectra matching to a database containing in silico or empirically generated peptide fragmentation patterns. A similarity score will be calculated to assign peptide-spectrum match (PSM) typically with a false discovery rate (FDR) controlled below 1% by a “target-decoy” approach [25]. Not all peptides can be detected by MS due to differences in their physicochemical properties, abundance, and ionization efficiency typically leading to a median sequence coverage of around 30% in tissue proteomes [26]. Consequently, proteins indistinguishable from each other based on identified peptides are grouped to form a protein group. The major alternative workflow to “bottom-up” proteomics is “top-down” proteomics, in which intact proteins are introduced into and measured by LC-MS/MS without enzymatic digestion [27], in principle allowing different proteoforms derived from one protein-coding gene to be distinguished. However, experimental challenges render this approach so far not amenable to large-scale proteomics investigations. In contrast, state-of-the-art bottom-up proteomics routinely identifies more than 6000 protein groups in cells and tissues in single run analyses and more than 10,000 protein groups after fractionation [26,28,29]. Blood plasma has one of the most complex proteomes with a dynamic range of protein concentrations of more than 10 orders of magnitude with the top 22 proteins comprising already 99% of total protein mass [30,31]. Due to the high dynamic range of plasma proteome and limitations in sensitivity that mass spectrometers can currently reach, measuring all plasma proteins remains elusive. The human Plasma Proteome Database (PPD) contains more than 10,000 protein products corresponding to 3778 distinct protein-coding genes [30,32]. The largest human plasma proteome dataset generated in a single study to data contains over 5300 proteins by ‘super-depletion’, extensive fractionation, and isobaric labelling—corresponding to 5002 genes [33]. These deep plasma proteomes entail additional experimental steps such as peptide fractionation and depletion of high-abundant proteins. These approaches increase the overall analysis time per sample and introduce variability to the workflow and are thus not preferred for large-scale proteomics investigations in a clinical setting [17]. At the current state of the MS technology, cost and investment of time are still often prohibitive for such workflows, even if low abundant proteins could be detected. The throughput and proteome depth of a given study have to be balanced depending on the budget and scope of the study. Depending on the LC-MS/MS instruments and acquisition methods used, current high-throughput methods, potentially applicable in the clinics, enable routine analysis of 30–60 plasma samples per day without depletion or pre-fractionation with a depth of 300–500 protein groups in a single run [34,35,36]. 

### 2.2. Proteomics Platforms beyond MS

While high-throughput MS-based plasma proteomics workflow routinely quantifies hundreds of the top abundant proteins, non-MS-based platforms in principle offer the simultaneous detection of thousands of proteins in a plasma sample. These technologies include the SOMAscan assay [37,38] and the proximity extension assay (PEA) commercialized by Olink Biosciences. Both technologies rely on reagents binding to proteins of interest (chemically modified nucleotides in SOMAscan and oligonucleotide-labeled antibody-pairs in PEA) for the “identification”, and the amplification of reporter sequences by quantitative real-time PCR or DNA microarrays for the quantification [37,39]. These immunoaffinity-based platforms could serve as complementarity to MS-based proteomics for detecting low-abundant proteins that are difficult to detect by MS, such as the Olink Inflammation panel that targets 92 inflammation-related protein biomarkers. However, there are long-recognized limitations associated with antibodies and other binders such as nonspecific binding and cross-reactivity, particularly in a highly multiplexed setting. Besides, both SOMAscan and the PEA assay are optimized for body fluid samples, i.e., plasma and serum, and are not designed for binding sites with PTMs or peptide variants that impede the binding of reagents. 

MS-based proteomics has the advantage of specifically discovering and quantifying proteins in an untargeted manner, and is clearly the most powerful platform for analyzing tissue proteomes, PTMs, protein-protein interactions, and protein variants. In the case of plasma to solve the dynamic range issue, a recent trend is to combine multiple platforms to cover a broader range of proteins taking advantages of the complementary strengths of both targeted and untargeted approaches [40,41]. 

### 2.3. MS-Based Metabolomics and Lipidomics

Metabolomics is the study of metabolites broadly defined as non-peptide molecules of less than 1.5 kDa [42]. Lipidomics, as a subset of metabolomics, is dedicated to lipid analysis with tailored extraction protocols, analytical methods, and data analysis strategies [43,44,45,46]. The main polar compound classes in the human metabolome comprise carbohydrates, ketones, amino and other organic acids, as well as biogenic amides, whereas the hydrophobic ones, namely lipids, are grouped into eight categories, namely fatty acyls, glycerolipids, glycerophospholipids, sphingolipids, saccharolipids, polyketides, sterol and prenol lipids [47] (Table 1). Among these small molecules, bile acids are of particular interest in NASH given their potent roles in mediating metabolic functions [48], as illustrated by the fact that several agonists of the bile acid receptor—Farnesoid X receptor (FXR) and its downstream target FGF19 are in phase I and II trials in treating NASH [49,50,51]. The structural diversity of the human metabolome poses a major challenge for analytical methods [52] resulting in various analytical approaches suited for detecting different classes of small molecules based on MS: LC-MS, gas chromatography mass spectrometry (GC-MS), imaging mass spectrometry, capillary electrophoresis–mass spectrometry, nuclear magnetic resonance, and Fourier transform infrared spectroscopy [53,54]. MS is the most commonly applied technology in metabolomics for the possibility of structural elucidation based on MS/MS spectra and metabolite annotation with higher confidence [55]. Compared with GC, where sample derivatization is often required, LC-MS based workflows are advantageous in clinical research for easier sample preparation. Hence, in the following section, we have chosen to focus on LC-MS-based workflows applied in metabolomics and lipidomics. 

In a typical LC-MS-based metabolomics workflow, hydrophilic metabolites are extracted using solvents such as acetonitrile or methanol [56], followed by separation using reversed-phase LC with a C_18_ stationary phase or hydrophilic interaction LC (HILIC) prior to MS analysis [57]. In untargeted studies, mass analysis is typically performed via high-resolution, accurate mass MS instruments such as the Orbitrap or TOF analyzers [58,59,60]. Chromatographic peaks across samples are then detected and reported as a list of metabolic “features” for further statistical analysis. There are multiple commercial and freely accessible software packages for this, including MZmine [61], XCMS [62], MSDial [63], MetaboScape (Bruker Daltonics, Germany), and Compound Discoverer (Thermo, Germany). Annotation of detected features (metabolite identification) is done based on LC-MS related properties including accurate mass, retention time, tandem mass spectra, and recently ion mobility [64]. However, due to the enormous chemical diversity of possible isobaric and isomeric structures, the identification of metabolites and the elucidation of chemical structures remain challenging. To illustrate, searching the mass 181.07066 (glucose, M+H adduct) in the human metabolome database [65] even with a 5 ppm mass accuracy already yields 24 compounds, not including known unknowns (molecules that have previously been mass measured but not identified) as well as complete unknowns. Recent developments in bioinformatics aim at partially annotating unknown metabolites by comparing their tandem mass spectra to those of known ones existing in online databases [66,67,68,69,70].

Unlike hydrophilic metabolites, extraction of lipids from biological samples is typically done using highly apolar solvents, like chloroform and methyl tert-butyl ether (MTBE) following four most commonly used standardized methods [71,72,73,74,75]. MS analysis of lipid extracts is performed using either direct infusion (termed shotgun lipidomics) or in conjunction with LC [76]. In LC-MS-based approaches, reversed-phase analysis on C_18_ columns dominates, which separates lipid species of the same class based on the interaction of fatty acyl chains with the stationary phase. In contrast, HILIC mainly separates lipids by polar head groups. A recent trend is to integrate ion mobility spectrometry into conventional MS-based workflows [77], to separate ions in the gas phase by their size and shape, which can be advantageous in resolving isomers. We have recently demonstrated the benefits of trapped ion mobility spectrometry and a highly sensitive data acquisition method (PASEF) in generating comprehensive lipidomics profiles from a small sample amount equivalent to 10 µg of liver tissue per injection [64]. Feature detection in lipidomics is often performed using the same tools as the polar part of the metabolome, but lipid annotation is done using dedicated modules and separate software [78,79]. Despite the seemingly simple structure of lipids, annotation faces various challenges arising from the multitude of isomers due to the positioning of double bonds and acyl chains in the molecule. In addition, liver and plasma samples might also contain lipids of odd-chain fatty acids derived from food intake and bacterial products in the gastrointestinal tract [80]. 

## 3. Proteomics-Based Biomarker Discovery Studies in Liver Disease

Hundreds of proteomics-based biomarker discovery studies in liver disease have been reported during the past two decades. In a recent literature review, we observed a significant bias towards hepatocellular carcinoma (HCC) and viral hepatitis among all causes of liver diseases, with only a small fraction of studies focusing on NAFLD and alcohol-related liver disease (ALD) despite them being the most prevalent types of liver disease [87]. More than 200 different proteins potentially useful for the diagnosis, prognosis, and progression stratification in NAFLD have been reported, typically in the form of a list of dysregulated proteins [88]. However, these can be difficult to interpret for clinicians or researchers engaged in translational research. Only a few of these studies took a step further to demonstrate the predictive or discriminative power of proposed biomarkers by building machine learning-based classification models, often predicting only one type of pathological condition: fatty liver [89,90], and recently fibrosis [91]. In addition, currently proposed candidate biomarkers suffered from low reproducibility and robustness, demonstrated by only one overlapping protein—MET (hepatocyte growth factor receptor) in the proposed protein marker panels for fatty liver in the two above-mentioned studies using immunoaffinity-based proteomics platforms. Furthermore, simply diagnosing fatty liver does not help clinical decisions, which are more concerned with liver fibrosis, the strongest predictor of liver- and all cause-related mortality as well as hepatic inflammation, which reflects disease activity [92]. In a recent study, a 12-protein panel was identified using the SomaScan proteomics platform which can distinguish between fibrosis stages F0–1 and F2–4 in patients with NAFLD with an area under the Receiver Operating Characteristics curve (AUROC) of 0.74 [91]. 

Recent progress in MS-based proteomics has enabled the generation of large datasets in clinical studies, accompanied by increasingly reproducible results. In an early effort, we identified polymeric immunoglobulin receptor (PIGR) as a predictor of NAFLD independent of insulin resistance [36], and this association between PIGR and NAFLD was subsequently reproduced in other studies [35,93,94]. Even though the focus of this review is NAFLD, ALD is indistinguishable under the microscope in terms of histological features, and hence might share common biomarkers. In a more recent effort, we acquired plasma proteomes from close to 600 individuals of biopsy-verified ALD and healthy controls, as well as 79 liver proteomes from the disease group [35]. Among the major findings, we identified proteomic marker panels to predict significant liver fibrosis (AUROC = 0.88), mild inflammation (AUROC = 0.83), and any presence of steatosis (AUROC = 0.89) with superior or comparable performance compared to existing best-in-class clinical tests including the FibroScan, the M30 apoptosis marker for hepatic inflammation, and the CAP value for liver steatosis. By integrating proteome changes in paired liver- and plasma samples, we could attribute the tissue origins of many of the proposed candidate markers. Comparing with the previous NAFLD study, three proteins PIGR, ALDOB, and LGALS3BP were common and robust markers for NAFLD and ALD. Given a NAFLD study of equivalent size and patient heterogeneity, it is likely to identify more circulating markers common to NAFLD and ALD. Recently, PIGR was also reported to be upregulated in patients with COVID-19 infection [95], possibly indicating it might not be specific to liver disease but reflect a general inflammation process. In any case, based on current results, PIGR is an indicator of hepatic inflammation and liver fibrosis in the context of liver disease. Importantly, proteomics-based biomarker discovery allows the identification of not only one single protein but rather panels of proteins, which collectively reflect the complex nature of the disease pathology and the need to study it from a systems biology perspective [35]. 

## 4. Metabolomics-Based Biomarker Discovery Studies in NASH

To provide an overview of recent metabolomics studies in NASH, we systematically searched for publications in the PubMed database using the logic terms “(nonalcoholic steatohepatitis OR NASH OR non-alcoholic fatty liver disease OR NAFLD) AND (lipidomics OR metabolomics) AND (human OR clinical)” for the period from 1 September 2015 to 1 September 2020. In this review, we only considered original research articles, which use MS and human samples. High complexity of the liver metabolome has opened up various MS applications in biomarker discovery (Table 2), ranging from polar metabolites [96] to lipids [97] using both targeted [98] and increasingly popular untargeted approaches [99]. Most of the studies shown in Table 2 reported perturbations in triglycerides, amino acids, fatty acids, and basic mitochondrial energy metabolism in NASH/NAFLD. Due to the diverse changes associated with NAFLD/NASH across many classes of lipids and metabolites, there is no clear consensus among the studies on candidate biomarkers or biochemical pathways (Table 2). This is potentially due to the large inter-individual variations in the metabolome and its extremely dynamic nature. Having a separate validation cohort for the biological confirmation of newly identified biomarker signatures might help to avoid misinterpretation of any study outcome and achieve more reproducible findings. Making data publicly available can further promote reproducible and transparent research. Surprisingly, our review shows that only three out of the 25 reviewed publications validated their findings in a separate study [100,101,102]. Moreover, none of the 25 studies released data in a public repository for future meta-analyses, although an initiative to standardize the reporting of metabolomics studies has been formed years ago [103,104]. Nine of the 25 studies not only proposed potential biomarkers but also evaluated the classification performance. These proposed marker candidates are summarized in Table 3 together with other omics markers. In brief, sample sizes range from 31 to 1479 with five studies having a sample size of below 100. Only five studies validated the marker performance in a validation cohort, with sample sizes ranging from 22 to 192. Most of these studies focus on predicting NASH in NAFLD patients, with a few exceptions, which predict significant or advanced fibrosis in patients with NASH, or distinguish between NAFLD and healthy individuals [100,102,105,106]. Based on these studies, circulating metabolome has good predictive power in identifying NASH and fibrosis in patients with NAFLD, as well as distinguishing between patients with NAFLD and healthy individuals. With a logistic regression model based on a biomarker panel consisted of eight lipids, one amino acid, and one carbohydrate, the AUROC for identifying advanced fibrosis (F3–4) in NAFLD was 0.94 in the discovery cohort (n = 156) and 0.84 in the validation cohort (n = 142) [100]. In another study of a smaller cohort (n = 31), an AUROC of 1.0 was achieved in predicting significant fibrosis (F2–4) with a support vector machine based on a marker panel of 10 lipids including diglycerides, triglycerides, and (lyso)phosphatidylcholines [105]. However, a validation cohort was not provided. Using a panel of 11 triglycerides or a combination of 11 metabolite features and three clinical markers, an AUROC of 0.9 and 0.94 was achieved respectively in identifying patients with NAFLD against healthy individuals [102,106]. Similarly, modest to high performance was achieved in predicting NASH in patients with NAFLD with AUROCs ranging between 0.65 and 0.95 (Table 3). Agreements of the AUROC between discovery and validation cohorts are generally good, with extremes differing as much as 0.16 (worse in validation) [102]. Apart from the highly dynamic nature of the human circulating metabolome, a few additional factors may contribute to such huge discrepancy in model performance between discovery and validation cohorts including differences in the distribution of disease severity, over-fitting in model training, or underpowered study design. 

**Figure 1:**
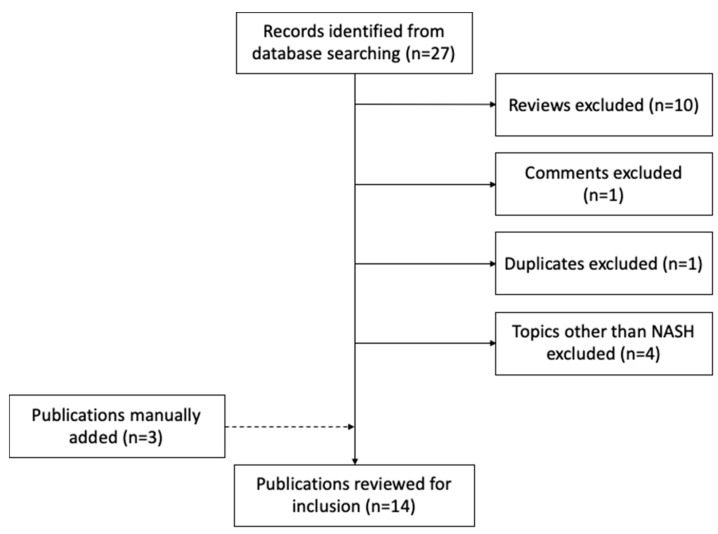
Flow chart of studies identified, excluded, and included.

Similar to metabolomics, we retrieved publications from PubMed database using the logic terms “(nonalcoholic steatohepatitis OR NASH OR non-alcoholic fatty liver disease OR NAFLD) AND (multiomics OR multi-omic)”, for the period from 1 September 2015 to 1 September 2020. This search strategy generated 27 records. We only considered articles that were not reviews or conference proceedings. The PubMed query did not retrieve three other relevant works, which we added manually. In total, this resulted in 14 papers meeting our criteria (Figure 1 and Table 4). We were first surprised by the small number of studies that have applied multi-omics techniques in this field so far. Although irrelevant to this review, replacing the keyword of “NASH” to “liver disease”, the search query resulted in 114 records, with a large proportion of studies focusing on hepatocellular carcinoma and other types of liver cancer. These search results implied limited resources of multi-omics datasets that have been generated on the topic of NASH, and a study bias towards liver cancer among all liver diseases, which is in concordance with a recent review on plasma proteomics efforts in liver disease [87]. Below, we describe the omics data types, research aims, experimental design, data integrative strategies, and study outcomes of the selected papers.

### 4.1. Characteristics of Studies

**Figure 2:**
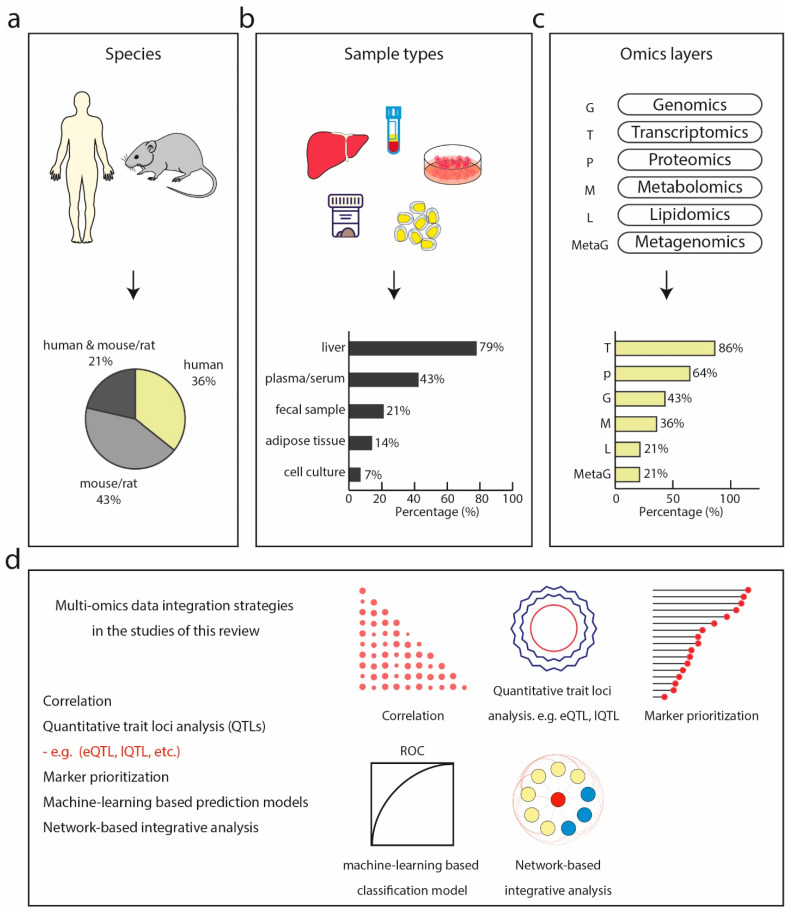
**Multi-omics studies in NASH.** (**a**) Species of which bio-specimens were used in the surveyed studies including human, model organisms such as mouse and rat, or a combination of both. (**b**) Sample types collected and analyzed by omics technologies in the surveyed studies, dominated by liver, followed by plasma or serum, fecal samples, adipose tissue, and cell culture. (**c**) Omics technologies used in the surveyed studies with transcriptomics being the most commonly used, followed by proteomics, genomics, metagenomics, lipidomics, and metagenomics. (**d**) Multi-omics integration strategies used in the surveyed studies.

Among these 14 studies, six characterized a specific biological or disease model using multi-omics datasets. For instance, a systemic approach was used to characterize the molecular alterations of a carbohydrate-restricted diet on hepatic steatosis in humans [124], and to describe the molecular profiles of a diet-induced obese model of NASH [125]. Only three studies focused on finding biomarkers or identifying discriminative molecular signatures for predicting fatty liver disease using multi-omics data [89,90,94]. These studies performed omics technologies on human (36%), mouse, or rat (43%) or a combination of both (21%) (Figure 2a). In terms of sample types, most of the studies used liver biopsies followed by blood plasma/serum, fecal samples, and adipose tissue of human and rodent origin (Figure 2b).

Transcriptomics was the most frequently performed (86% of all studies), followed by proteomics (64%) and genotyping (43%) (Figure 2c). Metabolome, lipidome, and metagenome were the least commonly generated data types, accounting for only 36%, 21%, and 21%, respectively. Transcriptomics and proteomics are most frequently combined. This could reflect to some extent the maturity, throughput, and accessibility of these technologies to non-specialized researchers. The majority of these studies generated new omics data along with the publications, however, only half of them made the data publicly accessible. The inaccessibility of publicly available datasets in turn hinders in silico-only studies. Most of the RNA sequencing data were made publicly available at the NCBI Gene Expression Omnibus and the NCBI Sequence Read Archive (SRA) database. Among the nine studies that included proteomics data, five used MS-based proteomics with the remaining adopting antibody-based approaches. Despite the growing consensus in the proteomics community about making mass spectrometry raw data accessible and reusable by uploading to a public database like PRIDE [135], only one study [94] did so (project identifier: PXD014751). In line with what we found in our above-described metabolomics review, only one study [126] deposited metabolomics data at the MetaboLights database (https://www.ebi.ac.uk/metabolights/), a database for metabolomics experiments maintained by the European Bioinformatics Institute (EMBO EBI). One study [130] deposited lipidomics mass spectrometry data at the Chorus project (http://chorusproject.org).

### 4.2. Overview of Data Integration Strategies

One of the advantages of applying multi-omics technologies to the same biological system is to understand the flow of information underlying disease and interpret the data in a holistic way in the context of biological networks and molecular interactions. Currently, omics data integration methods generally fall into two categories: multi-staged analysis and meta-dimensional analysis [136]. The difference between these two approaches is that multi-staged analysis performs data integration in a stepwise manner, adding one additional omics layer at a time, whereas meta-dimensional analysis attempts to incorporate and analyze all the types of data simultaneously. A systematic review of such existing tools can be found elsewhere [137]. In the surveyed literature, data integration was performed at different stages, predominantly at data analysis (data level, Table 4), followed by statistical and pathway data integration (result level, Table 4). Among those that perform integration at data level, various bioinformatics techniques were used, including machine learning-based approaches [89,90,134], correlation between two data types [129,130,134], quantitative trait loci (QTL) analysis [130,132], and network-based association analysis [132,133] for integrating more than one dataset, and weighted gene co-expression network analysis (WGCNA) [130] on a single layer of omics data (Figure 2d). Functional enrichment analysis including gene set enrichment analysis (GSEA) for KEGG pathways and GO terms were commonly employed in studies that integrate data at the level of statistical and bioinformatics results [125,127,128] (Supplemental Table S1). In one of them, the authors performed liver proteomics and metabolomics analysis to investigate the molecular mechanism underlying the Roundup pesticide in inducing liver pathology using a rat model [128]. By performing differential expression analysis followed by functional annotation using pathway analysis tools, the authors identified proteome changes associated with lipid detoxifying metabolic processes indicating lipid peroxidation, oxidative stress, and hepatocyte injury, all NASH-like pathological features. This association with a NASH-like phenotype was further supported in the metabolome profile by an increase in metabolites of oxidative stress and fibrosis markers. 

### 4.3. Multi-Omics Classifiers and Discriminative Disease Signatures

When the aim is to select predictive features for disease, machine learning approaches can treat multi-omics variables equally, also considering interaction between variables across omics layers. Three studies performed model-based integration at the data level to identify discriminative omics signatures for predicting disease phenotype [89,90,94]. Baseline data from the deep phenotyped IMI DIRECT cohorts (n = 1514) were used to build machine learning models for predicting NAFLD [89]. With a selected set of clinical and omics variables, a random forest machine learning model predicts NAFLD with an AUROC of 0.84, higher than those using only clinical data or any other omics data alone. Interestingly, when examining the predictive ability of each omics dataset as input variables alone, proteomic markers yielded the highest predictive accuracy surpassing genetic-, blood transcriptomics-, and metabolomics data. The proteomics data generated in this study derived from a combination of various immunoassays that target proteins with known associations to disease. Whether the use of an unbiased proteomics technology, i.e., MS-based proteomics, affects the predictive accuracy requires further investigation. In another biomarker discovery study, a multi-component classifier for NAFLD was developed, based on genotyping, serum proteomics, and clinical data such as plasma glucose level, HDL, and ALT [90]. The authors assessed the performance of classifiers based on each data domain alone and found that proteomics achieved the highest AUROC of 0.913, followed by phenomics data (0.886) and PNPLA3 genotyping data (0.596). Combining all markers selected from each individual data domain achieved an AUROC of 0.935. Similarly, in a biomarker discovery pre-clinical study, liver transcriptomics and proteomics as well as plasma proteomics were performed on a rat model of NASH aiming to characterize the molecular pathophysiology of NASH and to identify new plasma biomarkers [94]. By collecting molecular signals associated with NASH pathogenesis, the authors developed a multi-dimensional ranking approach integrating multi-omics data with liver histology characterization and prior knowledge and uncovered known as well as novel marker candidates of NASH and fibrosis. This study demonstrated that the integration of liver transcriptomics with liver- and plasma proteomics captured the translation of molecular changes from the diseased liver at the RNA level to the changes of liver and plasma protein level, and increased the biological resolution of discovered potential non-invasive biomarkers. Of the above-mentioned studies, only the one that utilized the IMI DIRECT data performed external validation using the UK biobank cohort on selected prediction models that were built on widely available clinical parameters.

## 5. Conclusions and Prospects

MS-based omics technologies are powerful tools to study human health and disease, and have a great potential to revolutionize tomorrow’s clinical laboratory diagnosis. Despite the extremely low translation rate of basic scientific findings into clinical applications in the early efforts, we are starting to see more reproducible and convincing results generated across clinical cohorts by independent research groups, especially in biomarker discovery studies in liver disease using MS-based proteomics. As clinical proteomics is increasingly capable of large-scale analysis of patient samples, machine learning-based approaches are emerging in large clinical studies to demonstrate the predictive power of newly identified composite marker panels. Looking forward, the FDA has already cleared a few MS-based devices for clinical use. However, as of today, no LC-MS-based diagnostic test that measures proteins or peptides has been approved. Apart from biological and clinical validation, a robust and quantitative proteomics assay needs to be established and validated across hospital sites and instruments to be used in the clinic. 

Existing clinical metabolomics and lipidomics studies in NASH have unveiled a broad range of changes in multiple classes of metabolites and lipids. A few studies have also identified potential biomarker panels for detecting different stages of fibrosis and NASH in NALFD. However, collectively they do not converge in terms of the core dysregulated metabolic pathways or potential biomarkers. As we have argued in the review, a well-designed clinical study including the use of a validation cohort, standardization of the experimental pipeline, and the potential release of the research data can help generate reproducible and robust results, further unlocking the real power of clinical metabolomics and lipidomics. Several pioneering studies have already integrated multi-omics data types generated on the same cohorts to build classifiers for detecting NAFLD, including genotyping, immunoaffinity-based proteomics, and MS-based metabolomics. Despite the minimal overlap among the proposed biomarker panels in previous literature, these newer studies clearly demonstrate the advantages of model performance when integrating multiple layers of omics information compared with using single layers of omics data alone. 

A common issue of omics-based biomarker discovery is the lack of classification performance of the proposed biomarkers, and the lack of verification in independent cohorts. Good practice in machine learning is necessary for training reliable, repeatable, and reproducible models [138]. In general, external validation in independent cohorts is always required to test the generalization ability of a learned model. From the surveyed literature, we have observed that there is a moderate to good agreement in the predictive power of candidate markers between discovery and validation cohorts. However, some studies also show great discrepancies. As we inferred, this may be due to differences in disease severity distribution, poor or insufficiently robust technical workflows for generating omics data, overfitting during model training, or underpowered study design. Considering these elements during study design will increase the success rate in future biomarker discovery studies and the subsequent implementation in clinical practice. Depending on the performance evaluation strategy and the disease severity distribution of the study population, it may be difficult to compare model performance across studies. This should also be taken into consideration when evaluating performance of emerging markers, especially across platforms. As more and more data are generated in clinical studies of NASH/NAFLD, it is promising to develop a powerful composite marker panel based on omics to detect disease. In addition to improving predictive power, compared with traditional markers that usually focus on a single aspect of the disease, multi-omics composite biomarker panels may also capture more biological complexity of disease pathogenesis and progression. However, if omics-based marker panels only provide marginal gain in terms of diagnostic performance compared to the best performing omics data type, practically it may be preferred to develop a diagnostic test based on a single technology. We believe that future research should focus on identifying diagnostic markers that can detect early stages of fibrosis and NASH in high-risk populations, such as individuals with obesity or type 2 diabetes. In addition, only a small percentage of patients progress from simple steatosis to NASH. Such predictive markers of can also benefit the clinical management of disease progression. We predict that prospective longitudinal studies to identify omics-based predictors of disease progression and therapeutic response will help to provide an alternative to liver biopsy, thereby avoiding unnecessary invasive testing and expediting drug development. In addition, the integration of omics datasets through powerful computational methods will help infer causality and reveal new insights into disease mechanisms. Finally, image based spatial omics provides unique opportunities to study the molecular profile of tissue sections at the level of single cells and organelles. In spatial metabolomics in particular, it has become possible to localize metabolites, lipids, and drugs in tissue sections through imaging mass spectrometry [139]. Although spatial proteomics and metabolomics are emerging fields, they will be a very valuable addition to research in liver diseases.

## Figures and Tables

**Table 1:** Overview of proteomics, LC-MS based metabolomics, and lipidomics platforms.

Aim of the Analysis	Proteomics	Proteomics	Proteomics	Metabolomics	Lipidomics
Platform	Mass spectrometry	Proximity Extension Assay (Olink)	Aptamer-based platform (SomaScan)	Mass spectrometry	Mass spectrometry
Analytes	Proteins	Proteins	Proteins	Polar small molecules (below 1500 Da), including carbohydrates, ketones, aminoacids andbiogenic amides	Apolar molecules, including glycerolipids, glycerophospholipids, sphingolipids, saccharolipids, polyketides, sterol and prenol lipids [47]
Analyte separation	Liquid chromatography	No	No	Liquid chromatography	Liquid chromatography (no for direct infusion)
Analysis of body fluids (e.g., plasma, serum, urine, saliva)	Yes	Yes	Yes	Yes	Yes
Analysis of tissue/cell culture	Yes	Not optimal	Not optimal	Yes	Yes
Analysis of proteoforms (PTMs, isoforms, coding-variants)	Yes	No	No	Not applicable	Not applicable
Analyte multi-plexing	Varies depending on the workflow and specimen, up to >10,000 proteins [29,81] (300–5000 in plasma) [33,35,36]	1472 proteins (targeted panels of 48–384 proteins) ^a^	7000 proteins ^b^	Varies depending on the workflow and specimen [82] (expected up to 10% of molecular features identification) [83]	Varies depending on the workflow and specimen [82], currently up to 1108 lipids in plasma [64]
Dynamic range	High-medium abundance in case of plasma	(pg/mL to µg/mL) ^c^	(femtomolar to micromolar) ^d^	(picomolar to milimolar) [42,84]	(picomolar to nanomolar) [85]
Reproducibility	Medium	Medium to High (Intra-assay CV 10–12% for the 384-protein panels) ^e^	High (Median intra-assay CV 4.6% for the 1300-protein platform) ^f^	Medium to high [83,86]	Medium to high [86]
Throughput (per day per system)	Low to medium (10 s–100 s)	High (100 s)	High (100 s)	Medium to high (10 s–100 s)	Medium to high (10 s–100 s)
Quantification	Relative or absolute for targeted assays	Relative or absolute for targeted assays	Relative	Relative or absolute for targeted assays	Relative or absolute for targeted assays

^a^https://www.olink.com/products/olink-explore/; ^b^https://somalogic.com/life-sciences/; ^c^https://www.olink.com/content/uploads/2017/02/1068-v1.0-Proseek-Multiplex-Development-Validation-data_final.pdf; ^d^https://somalogic.com/dynamic-range/; ^e^https://www.olink.com/content/uploads/2021/03/Explore_Demo_Certificate-of-Analysis_2021-03-30.pdf; ^f^https://mohanlab.bme.uh.edu/wp-content/uploads/2017/02/SSM-002-Rev-4-SOMAscan-Technical-White-Paper.pdf. All the above webpages were last accessed on 1 October 2021.

**Table 2:** Overview of MS-based metabolomics in NASH/NAFLD human studies.

Study Number	Study Aim	Sample Type	Data Release	Omics Type	Analytical Method	Metabolic Alterations	Reference
1	D	liver	NA	UL	LC-MS	TG, FA, CER	(Luukkonen et al., 2016) [107]
2	D	serum	NA	TM	LC-MS	AA, TCA	(Sookoian et al., 2016) [108]
3	D	plasma	NA	UM	LC-MS	AA	(Jin et al., 2016) [109]
4	D	serum	NA	UM	LC-MS	PL, PN, PTA, CE, I	(Tan et al., 2016) [110]
5	DV	serum	NA	UM	LC-MS	AA, OA, B, CR, PC, lyso-PC	(Chen et al., 2016) [101]
6	D	serum	NA	TM, TL	LC-MS	Lyso-PC, PC, AA, SM	(Feldman et al., 2017) [111]
7	DV	plasma	NA	UM, UL	LC-MS, GC-MS	AA, lyso-PC, PE	(Zhou et al.,, 2016) [112]
8	D	serum, liver	NA	UL	LC-MS, GC-MS	FA, TG, PC	(Luukkonen et al., 2017) [97]
9	D	serum	NA	UM, UL	LC-MS	TG, DG, FA, CER	(Alonso et al., 2017) [99]
10	D	liver	NA	UL	LC-MS	PS, TG, CER, PE, PC, PI, SM, CE, DG, FA	(Chiappini et al., 2017) [113]
11	D	urine	NA	UM	LC-MS	NAC, AA	(Dong et al., 2017) [114]
12	D	urine	NA	UM	GC-MS	G; AAD; X	(Troisi et al., 2017) [115]
13	D	RBC	UR	TL	GC-MS	FA	(Notarnicola et al., 2017) [116]
14	D	serum	NA	UM	LC-MS	AAD	(Qi et al., 2017) [117]
15	D	serum	NA	TM, TL	LC-MS	AA, CE, SM, CER, GPC	(Papandreou et al., 2017) [118]
16	D	serum	NA	UL	LC-MS	TG	(Yang et al., 2017) [119]
17	D	serum	NA	TL	LC-MS	NS	(Hu et al., 2018) [120]
18	DV	serum	NA	UM, UL	LC-MS	TG	(Mayo et al., 2018) [102]
19	D	serum	NA	UL	LC-MS	PC, SM	(Tiwari-Heckler et al., 2018) [121]
20	D	plasma, liver	NA	UL	LC-MS	PC, CL, CoQ, ACR	(Peng et al., 2018) [122]
21	DV	serum	NA	UM	LC-MS, GC-MS	AA, PT, FA, BA, ST	(Caussy et al., 2018) [100]
22	D	serum	NA	UM	LC-MS	AA, PC, UR	(de Mello et al., 2020) [123]
23	D	plasma	NA	UM	LC-MS	AA, lyso-PC	(Khusial et al., 2019) [106]
24	D	liver	NA	TM	LC-MS	RPD	(Zhong et al., 2019) [98]
25	D	serum	NA	UL, TM	LC-MS, GC-MS	DG, PC, PG, SM, PE, FA, GL	(Perakakis et al., 2019) [105]

D: discovery; V: validation; NA: no information available; UR: data available upon request; U: untargeted; T: targeted; M: metabolomics; L: lipidomics; LC-MS: liquid chromatography-mass spectrometry; GC-MS: gas chromatography-mass spectrometry; RBC: red blood cells; TG: triglycerides, FA: free fatty acids; DCE: dihydroceramides; CER: ceramides; TCA: Krebs cycle; PL: phospholipase; PN: purine nucleotide; PTA: phosphatidic acid; CE: cholesterol ester; I: inosine; OA: oleamide; B: bilirubin; PC: phosphatidylcholines; CR: carnitines; SM: sphingomyelin; PE: phosphoethanolamine; DG: diglycerides; PI: phosphatidylinositols; PS: phosphatidylserines; NAC: nucleic acids; AAD: amino acids derivatives; G: glucose; X: xylitol; GP: glycerophosphocholines; NS: nothing significant in omics data; CL: cardiolipin, CoQ: ubiquinone; ACR: acylcarnitine; PT: pentose; BA: bile acids; ST: steroids; UR: uridine; RPD: retinoid derivatives; PG: phosphatidylglycerol; GL: glycosMulti-omic studies in NASH.

**Table 3:** Emerging omics markers and their classification performance for diagnosing NAFLD/NASH.

Platform	Sample Type	Number of Analytes Quantified in Total	Sample Size (Discovery Cohort)	Sample Size (Validation Cohort)	Classifier	Prediction Target	Markers	AUROC	Reference
Omics	Serum	1129 proteins (SomaScan), 1 genotype, >200 clinical variables	n = 443	n = 133	Logistic regression	Hepatic steatosis in obesity	8 proteins + 1 genotype + 12 clinical variables: ACY1, SHBG, CTSZ, MET, GSN, LGALS3BP, CHL1, SERPINC1, PNPLA3 variant.	0.935 (0.914 in validation cohort)	(Wood et al., 2017) [90]
Omics	Serum	860 proteins, 288 metabolites, 108 SNPs, 16,209 protein-coding genes, 58 clinical variables	n = 1049	No for the omics model	Random forest	Fatty liver	185 clinical and omics features	0.84	(Atabaki-Pasdar et al., 2020) [89]
SOMAscan proteomics	serum	1305 proteins	n = 113	n = 71, n = 32	Elastic-Net	Fibrosis F0–1 against F2–4	serum amyloid P, fibrinogen, olfactomedin, and SHBG	0.74 (0.52–0.78 in validation cohorts)	(Luo et al., 2021) [91]
SOMAscan proteomics	serum	1305 proteins	n = 113	n = 71, n = 32	Elastic-Net	Fibrosis (F3–4 against F0–2)	latent transforming growth factor beta binding protein 4, IGF-1, vascular cell adhesion molecule 1, interleukin-1 soluble receptor type-1, IL18BP, thrombospondin-2, collectin kidney 1, SHBG, interleukin-27 receptor subunit alpha, leukemia inhibitory factor receptor, soluble, fibulin-3, and plexin-B2	0.83 (0.74–0.78 in validation cohorts)	(Luo et al., 2021) [91]
MS-based proteomics	Plasma	235–277 proteins	n = 19	NA	Unclear	Fibrosis F2–4 against F0–1	Complement component C7, α-2-macroglobulin, Fibulin-1, Complement component C8 γ chain; α-1-antichymotrypsin	0.79–1 for each individual protein	(Hou et al., 2020) [93]
Metabolomics	serum	365 lipids, 61 glycans and 23 fatty acids	n = 31	NA	support vector machine	Fibrosis F2–4 against F0–1	10 lipids: DG(36:3), LPC(18:0), PC(36:2), PC(37:2), PC(40:5), TG(38:0), TG(50:0), TG(51:1), TG(57:1), TG(60:2)	1	(Perakakis et al., 2019) [105]
Metabolomics	Serum	365 lipids, 61 glycans and 23 fatty acids	n = 80	NA	Support vector machine	NASH vs. NAFL vs. Healthy	29 lipids: AcCa(10:0), Cer(d34:2), DG(34:1), DG(36:4), LPC(20:0e), LPC(22:5), LPE(16:0), PC(32:0), PC(32:1e), PC(34:0), PC(34:2e), PC(35:3), PC(36:4), PC(36:5e), PC(37:2), PC(40:6e), PC(40:7), PC(40:8), PC(42:6), PE(38:1), PE(38:6), PI(36:1), SM(d32:0), SM(d32:2), SM(d40:1), TG(38:0), TG(38:2), TG(43:1), TG(53:5)	0.94–0.99 (one vs. rest)	(Perakakis et al., 2019) [105].
Metabolomics	Plasma	13,008 metabolic features	n = 559	NA	Random forest	NAFLD vs. non-NAFLD	11 metabolite features + 3 clinical variables: serine, leucine/isoleucine, tryptophan, three putatively annotated compounds, two unknowns, lysoPE(20:0), lysoPC(18:1), WC, WBISI, and triglycerides	0.94	(Khusial et al., 2019) [106]
Metabolomics	Serum	652 metabolites	n = 156	n = 142	Logistic regression	Fibrosis F3–4 vs. F0–2 in NAFLD	8 lipids + 1 amino acid + 1 carbohydrate: 5alpha-androstan-3beta monosulfate, pregnanediol-3-glucuronide, androsterone sulfate, epiandrosterone sulfate, palmitoleate, dehydroisoandrosterone sulfate, 5alpha-androstan-3beta disulfate, glycocholate, taurine, fucose	0.94 (0.84–0.94 in validation cohort)	(Caussy et al., 2019) [100]
Metabolomics	Serum	540 lipids and amino acids	n = 467	n = 192	Logistic regression	NAFLD vs. Healthy	11 triglycerides	0.9 (0.88 in validation cohort)	(Mayo et al., 2018) [102]
Metabolomics	Serum	540 lipids and amino acids	n = 467	n = 192	Logistic regression	NASH against NAFL	20 triglycerides	0.95 (0.79 in validation cohort)	(Mayo et al., 2018) [102]
Metabolomics	Serum	Sphingolipids and branched fatty acid esters of hydroxy fatty acids	n = 1479	NA	Logistic regression		oleic acid-hydroxy oleic acid	0.61	(Hu et al., 2018) [120]
Metabolomics	Serum	1761 metabolic features	n = 59	NA	Unclear	NASH against NAFL	pyroglutamate	0.846	(Qi et al., 2017) [117]
Metabolomics	Urine	Unclear	n = 78	NA	Unclear	NASH against NAFL	Pyroglutamic acid	0.65	(Dong et al., 2017) [114]
Metabolomics	Serum	Unclear	n = 223	n = 95	Logistic regression	NASH against non-NASH	glutamate, isoleucine, glycine, lysophosphatidylcholine 16:0, phosphoethanolamine 40:6, AST, and fasting insulin	0.882 (0.856 in validation cohort)	(Zhou et al., 2016) [112]
Lipidomics	Serum	239 lipids	n = 42	n = 22	Logistic regression	NASH in NAFLD	Monounsaturated triglycerol	0.83 in both discovery and validation cohorts	(Yang et al., 2017) [119]

**Table 4:** Overview of multi-omics studies in NASH/NAFLD.

Study Aim	Sample Type	Species	New Data	Data Release	Sample Size	Omics Integration	G	T	P	M	L	MetaG	Reference
C	Liver, serum, fecal samples	Human	Y	Y	n = 10, 7	Data	Y	Y		Y	Y	Y	(Mardinoglu et al., 2018) [124]
C	liver	Mice	Y		n = 9 for TP, n = 6 for scRNAseq	Data and results		Y	Y				(Ægidius et al., 2020) [125]
C	Liver, serum	Human	Y	Y	n = 18 for plasma, n = 9 for liver T			Y	Y	Y			(Wruck et al., 2015) [126]
C	Cell culture	Human	Y		n = 20	Results		Y		Y			(Mesnage et al., 2018) [127]
C	Liver	Rat	Y		n = 10, 10	Results			Y	Y			(Mesnage et al., 2017) [128]
C	Liver, plasma, feces	Mice, human	Y	Y	n = 10 in mice, n = 14 in patients	Data		Y			Y	Y	(Qian et al., 2020) [129]
B	Serum	Human	Y		n = 795 for T2D, n = 2234 for high risk of T2D	Data	Y	Y	Y	Y		Y	(Atabaki-Pasdar et al., 2020) [89]
B	Liver, plasma	Human	Y		n = 576	Data	Y		Y				(Wood et al., 2017) [90]
B	Liver	Mice	Y	Y	n = 48 for liver, n = 16 for plasma	Data and results		Y	Y				(Veyel et al., 2020) [94]
C	Liver	Mice	Y	Y	n = 385	Data	Y	Y	Y				(Jha et al., 2018) [130]
C	Liver	Human, mice				Data		Y	Y				(Lee et al., 2017) [131]
C	Liver, adipose tissue	Mice	Y	Y	n = 228 from 113 mouse strains	Data	Y	Y					(Krishnan et al., 2018) [132]
C	Liver, adipose tissue	Mice				Data	Y	Y					(Kurt et al., 2018) [133]
C	Liver	Human, mice	Y	Y	n = 144 in human, n = 6 in mice	Data and results		Y	Y				(Xiong et al., 2019) [134]

C: characterization; B: biomarker discovery; G: genotyping; T: transcriptomics; P: proteomics; M: metabolomics; L: lipidomics; M: metagenomics. Y: yes.

## Supplementary Materials

The following are available online at https://www.mdpi.com/article/10.3390/jcm10204673/s1, Table S1: Metadata of the reviewed literature.
